# Identifying potential key metabolic pathways and biomarkers in glaucoma: a systematic review and meta-analysis

**DOI:** 10.1136/bmjophth-2024-002103

**Published:** 2025-03-13

**Authors:** Navid Golpour, Rune L Brautaset, Flora Hui, Maria Nilsson, Jonas E Svensson, Pete A Williams, James R Tribble

**Affiliations:** 1 Department of Clinical Neuroscience, Division of Eye and Vision, St. Erik Eye Hospital, Karolinska Institutet, Stockholm, Sweden; 2 Centre for Eye Research Australia, Royal Victorian Eye and Ear Hospital, Melbourne, Victoria, Australia; 3 Department of Clinical Neuroscience, Karolinska Institutet and Stockholm Health Care Services, Region Stockholm, Stockholm, Sweden

**Keywords:** Glaucoma, Tears, Aqueous humour, Oxidative Stress

## Abstract

**Background:**

Glaucoma, a leading cause of irreversible blindness worldwide, is characterised by retinal ganglion cell degeneration. Increasing evidence points to metabolic dysfunction, particularly mitochondrial dysfunction, as a contributing factor to glaucomatous neurodegeneration. This systematic review and meta-analysis aimed to identify key metabolic pathways and biomarkers associated with primary open-angle glaucoma (POAG).

**Methods:**

A systematic literature search was conducted to identify studies measuring metabolites in plasma and aqueous humour from patients with POAG using metabolomics techniques. Enrichment analyses for significantly increased metabolites were conducted using MetaboAnalyst. Meta-analyses were performed using random-effects models to calculate effect sizes for metabolites reported in at least three studies.

**Results:**

17 studies involving patients with POAG were included. Pathway analysis revealed significant enrichment of the arginine and proline metabolism pathway in both aqueous humour and plasma. Additionally, the phenylalanine metabolism pathway was enriched in plasma. These pathways are associated with oxidative stress and neurodegeneration, both of which are key factors in POAG pathology. Meta-analysis identified several significantly elevated metabolites, including lysine, glutamine, alanine, histidine, carnitine and creatinine in aqueous humour, as well as methionine in plasma.

**Conclusions:**

This study underscores the central role of metabolic dysfunction in POAG, highlighting specific metabolites and pathways that could serve as biomarkers for early diagnosis and therapeutic intervention. Future research should prioritise longitudinal studies and untargeted metabolomic profiling to further deepen our understanding of metabolic changes and their contributions to glaucoma progression.

**PROSPERO registration number:**

CRD42024512098.

**WHAT IS ALREADY KNOWN ON THIS TOPIC**

- Metabolic dysfunction and oxidative stress are key contributors to primary open-angle glaucoma (POAG), but the metabolomic mechanisms remain poorly understood.

**WHAT THIS STUDY ADDS**

- It identifies previously unknown metabolic alterations in POAG, including enriched pathways and elevated metabolites in aqueous humour and plasma.

**HOW THIS STUDY MIGHT AFFECT RESEARCH, PRACTICE OR POLICY**

- These findings highlight metabolic dysfunction as a critical factor in POAG, paving the way for novel biomarkers and targeted therapeutic strategies.

## Introduction

 Glaucoma is the most common cause of irreversible blindness worldwide.^1^ This group of neurodegenerative diseases is characterised by progressive dysfunction and loss of retinal ganglion cells (RGCs) and their axons in the optic nerve. In the early phase visual loss is functionally compensated for by the overlapping visual fields between the two eyes, and cortical infilling,^2^ leaving the patient unaware of the vision loss. Additionally, there is a lack of cost-effective screening methods for early diagnosis.^3^ Diagnosis relies on a combination of clinical tests, including intraocular pressure (IOP), perimetry, gonioscopy and optical coherence tomography.^4^ None of these parameters alone offers high sensitivity or specificity for detecting glaucoma and need to be combined. There are no definitive biomarkers, and by the time symptoms appear, significant RGC loss has usually occurred.

Glaucoma risk factors include ageing, genetics and elevated IOP.^5^ While preclinical studies and clinical trials on neuroprotective strategies show promise, no treatments are available yet.^6^ The only validated treatment is lowering IOP through medication, laser or surgery.^7^ Despite successful IOP reduction, some patients continue to experience progression despite having normal IOP,^8^ suggesting that neurodegeneration in glaucoma is multifactorial and influenced by other factors. There is a pressing need to better understand these mechanisms to develop new treatments.

Increasing data indicate metabolic dysfunction, particularly of the mitochondria, as a key factor in neurodegeneration in glaucoma. The unmyelinated axons of the RGCs at the lamina cribrosa in the optic nerve head (ONH) are a primary site of RGC damage in glaucoma. Mitochondrial density is highest at this region.^9^ RGCs are highly metabolically active,^10^ with the bulk of their ATP generated via mitochondrial oxidative phosphorylation (OXPHOS), making them susceptible to mitochondrial abnormalities. OXPHOS dysfunction and mitochondrial DNA damage are two hallmarks of mitochondrial dysfunction in glaucoma.^11^ The resulting decrease in ATP production and increase in reactive oxygen species (ROS) contribute to the oxidative stress that drives neuronal degeneration in glaucoma.^12^ Underscoring the importance of mitochondrial function to RGCs is the fact that mitochondrial dysfunction is implicated in several other neurodegenerative diseases which primarily affect RGCs, such as in Leber hereditary optic neuropathy^13^ and autosomal dominant optic atrophy.^14^ Given the critical role of mitochondrial function for RGC survival, understanding metabolic alterations is important for revealing the mechanisms of glaucomatous neurodegeneration.

Metabolomics identifies and measures all the metabolites in an organism, which reflects its biochemical activity as by-products of cellular processes.^15^ Advances in techniques, such as nuclear magnetic resonance (NMR), gas or liquid chromatography-mass spectrometry (GC/LC-MS), have improved our understanding of the human metabolome by allowing large-scale mapping of hundreds to thousands of metabolites in a sample.^16^

Since the first meta-analysis on the metabolomics of patients with primary open-angle glaucoma (POAG) in 2022,^17^ the number of metabolomics studies of glaucoma has substantially increased.^18^ Given this, we present an updated meta-analysis which summarises the metabolic profiles in patients with glaucoma to identify metabolites and pathways reproducibly associated with glaucoma.

## Materials and methods

This systematic review and meta-analysis followed the Preferred Reporting Items for Systematic Reviews and Meta-Analyses (PRISMA) guidelines. The review protocol was registered with the International Prospective Register of Systematic Reviews in April 2023.

### Search strategy

A literature search was performed in the following databases: Medline (Ovid), Embase (Embase.com) and Web of Science Core Collection (Clarivate). The last search was conducted on 15 March 2024. The search strategy was developed in Medline (Ovid) in collaboration with librarians at the Karolinska Institutet University Library. For each search concept, Medical Subject Headings (MeSH terms) and free text terms were identified. The search was then translated into the other databases. Databases were searched from inception, and language was restricted to English. De-duplication was done using the method described by Bramer *et al*.^1^ The full search strategies for all databases are available in the online supplemental materials.

### Inclusion and exclusion criteria

Inclusion criteria were studies that (1) measured metabolites from plasma, serum, aqueous humour or tear film, (2) used metabolomics techniques and (3) reported on both patients with primary glaucoma and non-glaucomatous controls. Exclusion criteria were studies with (1) insufficient raw data, (2) no control group, (3) non-human studies, (4) single metabolites or biased panels, (5) duplicate data, (6) secondary glaucoma and (7) non-original articles (eg, editorials, reviews). These criteria were applied for both the systematic review and meta-analysis.

### Study selection

**Figure 1:**
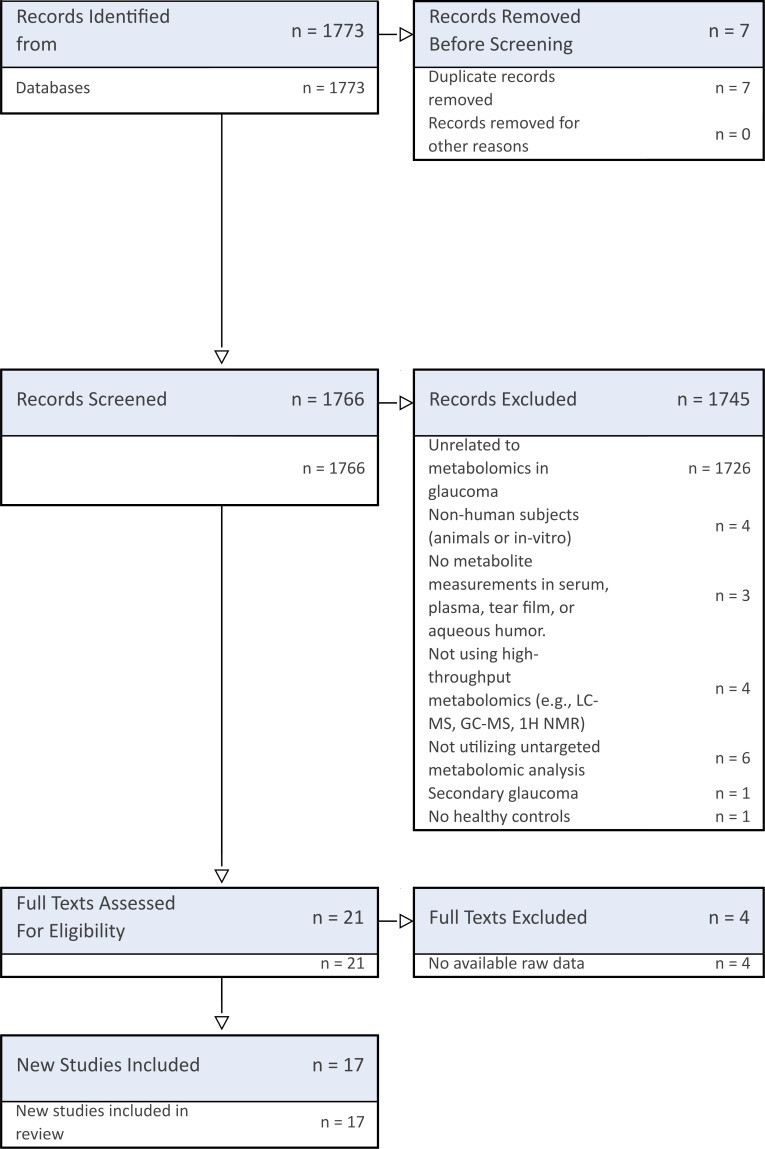
PRISMA flow diagram detailing study selection process. PRISMA, Preferred Reporting Items for Systematic Review and Meta-Analysis.

All retrieved articles were imported into DistillerSR for assessment. NG and JRT independently screened all the article titles, abstracts and full texts with PAW resolving any disagreements. Eligible studies underwent data extraction, as shown in the PRISMA flow diagram (figure 1). Any missing data were requested at least two times from the main author of the article, with a 1 month interval between requests. If the data could not be obtained, the study was excluded.

### Risk of bias in individual studies

The risk of bias and methodological quality of the included studies were assessed using the QUADOMICS tool, an adaptation of QUADAS designed specifically for -omics studies (online supplemental table 1). The tool comprises 16 questions, and responses for each study are summarised as a ratio. As the QUADOMICS tool lacks established thresholds, none were applied in this review.

### Data extraction

Data from each included study were extracted into a standardised Microsoft Excel spreadsheet, including the following:

Study details: authors, publication year, geographic location, sample size, sample type, glaucoma type, control group type, metabolomics technique and analytical approach (untargeted or semi-targeted).

Study results: identified metabolites with corresponding Human Metabolome Database IDs, concentrations (mean or median with IQRs or SD), fold changes (FCs), p values or adjusted p values (q values) and the total number of detected metabolites.

### Metabolic pathway analysis

Metabolic pathway analysis was conducted using MetaboAnalyst 5.0 and the Kyoto Encyclopedia of Genes and Genomes (KEGG) database to assess the functional impact of POAG on metabolism. Statistically significant metabolites (p<0.05) in plasma and aqueous humour were included. Enrichment analysis, using the hypergeometric test against KEGG pathways, was evaluated with both p values and q values, with significance set at <0.05. Topology analysis used relative betweenness centrality, considering pathways with an impact score ≥0.1 as significantly impacted.

### Meta-analysis of metabolites

Metabolites reported in at least three studies were included in the meta-analysis. Effect sizes (log(OR)) and SEs were pooled, with results presented as forest plots showing ORs and 95% CIs. Statistical significance was set at p<0.05. Analyses were conducted using Review Manager V.5.3, and heterogeneity was assessed with the χ² and I² statistics, following Cochrane guidelines. Heterogeneity classifications are detailed in online supplemental table 2.

### Effect size calculation

Effect sizes were converted to logarithmic fold changes log_2_(FC). The SE of the log_2_(FC) between glaucoma patients and controls was calculated for each metabolite. Specifically, the SE was determined by combining the corresponding SD $\left({SD}_{1}^{2}and{SD}_{2}^{2}\right)$, adjusted for their respective sample sizes (*n*_1_ and *n*_2_) and divided by the mean concentrations (*m*_1_ and *m*_2_). This method, described by Lajeunesse *et al*,^19^ is suitable for meta-analyses using response ratios in studies with correlated or multigroup designs. It was previously applied in the first metabolomics meta-analysis on POAG^17^ to standardise variance calculations for log-transformed response ratios. When the mean and SD were available, the SE of log_2_(FC) was directly calculated using Formula 1.

$$SE\left[{log}_{2}\left(FC\right)\right]=\;SE\left[\;{log}_{2}\left(\;\frac{m_{1}}{m_{2}}\;\right)\;\right]=\sqrt{\;\frac{SD_{1}^{2}}{n_{1}\;\times \;m_{1}^{2}}\;+\;\frac{SD_{2}^{2}}{n^{2\;}\times \;m_{2}^{2}}}$$

**Formula 1**. The SE of the log_2_(FC) was calculated using *m*_1_ and *m*_2_, *s*_1_ and *s*_2_ and SD_1_ and SD_2_.

If the median and IQR were reported, the data were assumed to be normally distributed. The median value was used in place of the mean, and the SD was estimated as $SD=\frac{IQR}{1.35}$. These estimates were then used in Formula 1 to calculate the SE of the log_2_(FC).

When only FCs and p values were available, the z-score corresponding to the reported p value was calculated. For one-sided tests, the p value was divided by 2 to obtain a two-sided p value. The SE of the log₂FC was then calculated using Formula 2:

$$SE\left[log\left(FC\right)\right]=\frac{log\left(FC\right)}{z}$$

**Formula 2**. Calculation of SE based on log_2_(FC) and *z*-value.

If adjusted p values (q values) were reported, they were converted to approximate p values using the Benjamini-Hochberg procedure in Formula 3 where i is the rank of the q value in the sorted list and *N* is the total number of detected metabolites. The SE of the log_2_(FC) was then calculated using Formula 2.

$$p=\frac{q\;\times \;i}{N}$$

**Formula 3**. Calculation of p value based on q value, rank (*i*) and total number of detected metabolites (*N*).

Metabolites were considered significantly altered if they met the criteria of p value <0.05 and, when available, q value (false discovery rate, FDR) <0.05. These metabolites were further classified as increased log_2_(FC) >1.0 or decreased log_2_(FC) <1.0.

## Results

### Literature search results

The database search yielded 1773 articles, of which 7 duplicates were removed, leaving 1766 for screening. After applying exclusion criteria, 1744 articles were discarded, primarily for being unrelated to metabolomics in glaucoma (n=1726). Of the 21 full-text articles assessed for eligibility, 4 were excluded due to unavailable raw data despite author requests. Finally, 17 studies met the inclusion criteria and were included in the meta-analysis (figure 1).

### Study characteristics

The 17 included studies are summarised in table 1. Eight analysed aqueous humour (AH), seven plasma, two serum and two both AH and plasma, while two analysed tear samples. We, therefore, did not consider there to be sufficient data from tear metabolomics studies with which to perform further analysis of the tear metabolome. All provided extractable quantitative data suitable for inclusion in the meta-analysis.

**Table 1:** Details of the study design for the included studies

No.	First author (year)	Country	Glaucoma type (n)	Control type (n)	Sample type	Technique
1	Barbosa Breda (2020)	Belgium	POAG (27), NTG (27)	Cataract (29)	AH	1 hour NMR
2	Botello-Marabotto (2024)	Spain	POAG (11)	Controls (19)	Tears	1H-NMR
3	Buisset (2019)	France	POAG (26)	Cataract (26)	AH	LC-MS
4	Kang (2022)	USA	XFG (205)	Controls (205)	Plasma	LC-MS
5	Nzoughet (2020)	France	POAG (34)	Cataract (30)	Plasma	LC-HRMS
6	Leruez (2018)	France	POAG (36)	Cataract (27)	Plasma	LC-MS/MS
7	Li (2024)	China	PACG (348)	Controls (268)	Serum	UPLC
8	Lillo (2022)	Spain	OAG (8)	Refractive error (16)	AH	LC-MS/MS
9	Myer (2020)	USA	POAG (16), PEX (31)	Controls (25)	AH	For POAG: 1H-NMR, For PEX: IROALC-MS
10	Pan (2020)	China	POAG (16)	Cataract (24)	AH	GC-TOF-MS
11	Pulukool (2021)	India	POAG (20)	Cataract (20)	AH	GC-TOF-MS
12	Rossi (2019)	Italy	POAG (16)	Controls (17)	Tears	LC-MS/MS
13	Tang (2021)	China	POAG (25)	Cataract (25)	AH+Plasma	LC-MS/MS
14	Zeleznik (2023)	USA	POAG (599)	Controls (599)	Plasma	LC-MS/MS+NMR
15	Gowtham (2023)	India	POAG (14)+PACG (14)	Cataract (14)	AH+Plasma	LC-HRMS
16	Gong (2020)	China	POAG (30)	Controls (30)	Serum	GC-MS
17	Burgess (2015)	USA	POAG (72)	Controls (72)	Plasma	LC-MS

Note: The complete reference list for table 1 is provided in online supplemental appendix AAppendix A ()..

AH, aqueous humour; GC-TOF-MS, gas chromatography–time-of-flight mass spectrometry; LC-HRMS, liquid chromatography–high-resolution mass spectrometry; LC-MS, liquid chromatography–mass spectrometry; NMRnuclear magnetic resonanceNTG, normal-tension glaucoma; PACGprimary angle-closure glaucomaPEXpseudoexfoliation syndromePOAG, primary open-angle glaucoma; UPLC, ultra-performance liquid chromatographyXFGexfoliation glaucoma

### Quality assessment

The risk of bias and methodological quality of the studies was assessed using the QUADOMICS tool, covering 16 criteria. Overall, 10 out of 17 studies achieved a perfect score, meeting all applicable criteria. The 7 studies that did not achieve perfect scores missed only one or two criteria. In general, the studies demonstrate high quality according to the QUADOMICS tool. The full assessment is available in online supplemental table 1.

### Metabolic pathways in patients with POAG

In this compiled data set, based on the results of the individual studies, levels of 33 metabolites in AH and 54 metabolites in plasma were significantly altered in patients with POAG compared with controls. The most frequently reported significant metabolites across both plasma and AH were hypoxanthine and arginine, each identified in five studies, followed by alanine, methionine and propionylcarnitine, each reported in four studies (online supplemental table 3).

**Figure 2:**
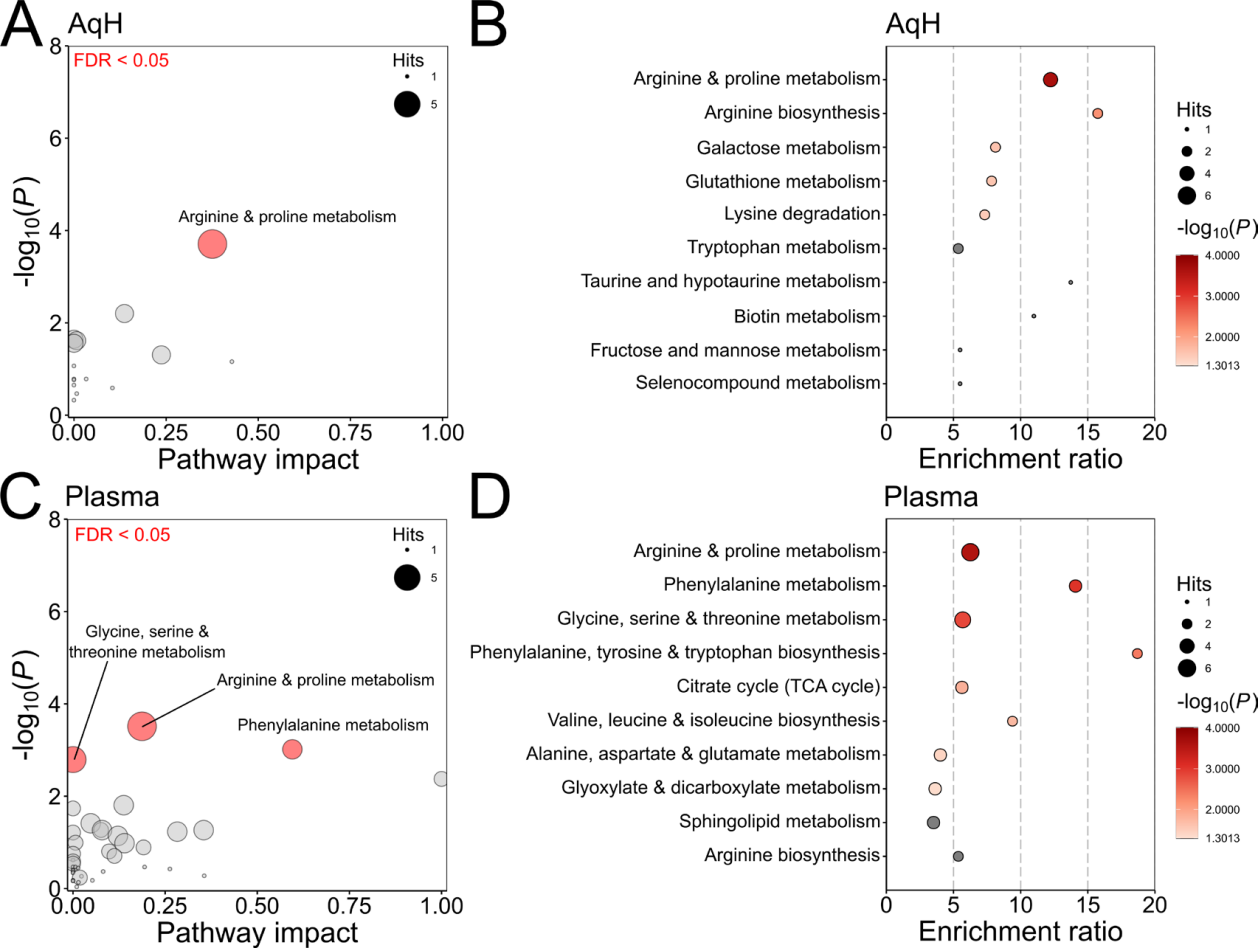
Pathway analysis (**A, C**) and enrichment analysis (**B, D**). Performed using MetaboAnalyst, based on 32 significantly elevated metabolites in the aqueous humour (**A, B**) and 70 significantly elevated metabolites in plasma (**C, D**) from patients with POAG compared with controls. All matched pathways are scaled by the number of pathway hits; significant pathways are shown in red. In both cases, arginine and proline metabolisms were significantly enriched, with phenylalanine metabolism additionally enriched in plasma. POAG, primary open-angle glaucoma.

All significantly elevated metabolites were subjected to pathway enrichment and impact analyses using MetaboAnalyst software. The analyses aimed to identify metabolic pathways that are potentially dysregulated in POAG (figure 2). After filtering based on p values, FDR and pathway impact scores, one significantly impacted pathway was identified in AH and two in plasma.

In AH, the arginine and proline metabolism pathway was significantly affected (pathway impact=0.376; p<0.05; FDR<0.05). In plasma, two pathways showed significant enrichment: arginine and proline metabolism (pathway impact=0.187) and phenylalanine metabolism (pathway impact=0.595). Additionally, the glycine, serine and threonine metabolism pathway had significant enrichment but had an impact value of 0 (figure 2). Complete results from the enrichment and pathway analyses, including all significant metabolic pathways and associated metabolites, are provided in online supplemental table 4.

### Metabolites in patients with POAG identified by meta-analysis

**Figure 3:**
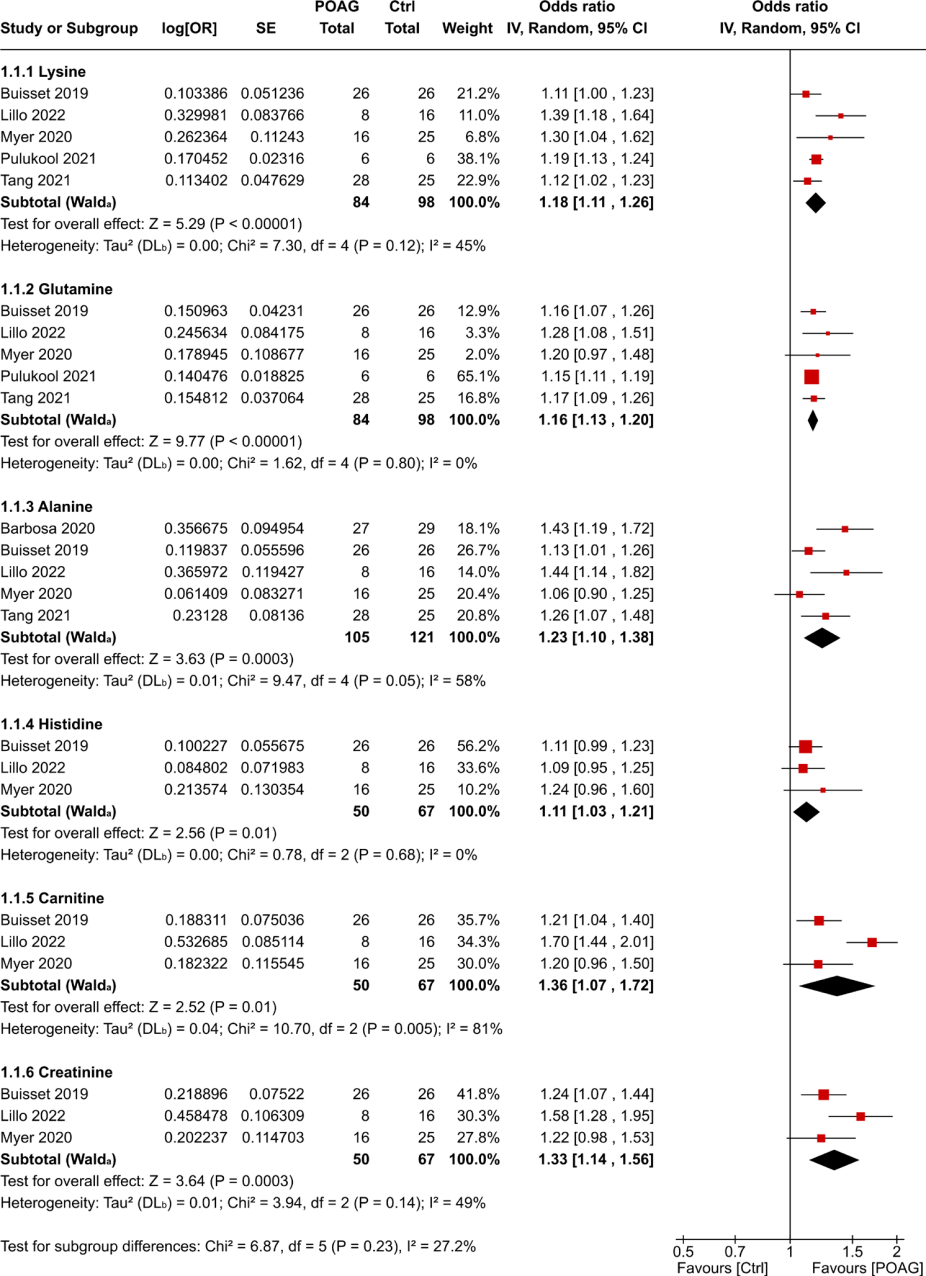
Forest plot of the statistically increased metabolites in aqueous humour of patients with POAG compared with control participants. Individual and pooled analysis presented as effect size (log(OR)) and CI 95% of lysine, glutamine, alanine, histidine, carnitine and creatine. POAG, primary open-angle glaucoma.

**Figure 4:**
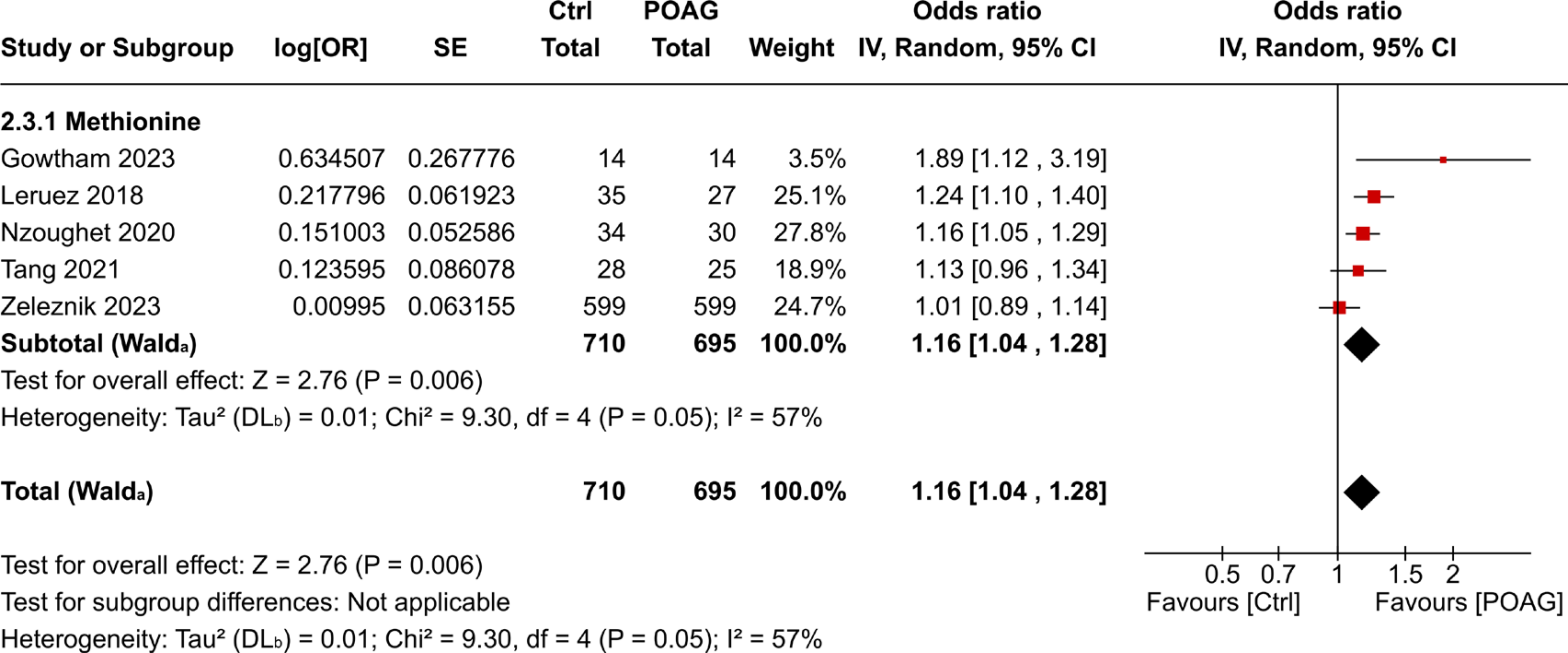
Forest plot of the statistically increased metabolites in plasma of patients with POAG compared with control participants. Individual and pooled analysis presented as effect size (log(OR)) and CI 95% of methionine. POAG, primary open-angle glaucoma.

A total of 19 metabolites in AH and eight metabolites in plasma, each reported in at least three independent datasets, were included in the quantitative meta-analysis. In AH, six metabolites were significantly elevated in patients with POAG compared with controls: lysine, glutamine, alanine, histidine, carnitine and creatinine (figure 3). In plasma, methionine was the only metabolite identified as significantly higher in patients with POAG compared with controls (figure 4). No metabolites were found to be significantly decreased in either AH or plasma. Metabolites that did not show significant changes are listed in online supplemental figures 1 and 2. Pathway analysis of the six AH metabolites that were significantly elevated did not demonstrate any significantly affected pathways, likely because of the small number of metabolites.

The degree of heterogeneity among the significant metabolites was assessed using the I² statistic. In AH, glutamine and histidine showed no heterogeneity (I²=0%), lysine (I²=45%), alanine (I²=58%) and creatinine (I²=49%) had moderate heterogeneity. Carnitine showed substantial heterogeneity (I²=81). In plasma, methionine demonstrated moderate heterogeneity (I²=57%).

No metabolites in the plasma or AH of patients with primary angle-closure glaucoma, exfoliation glaucoma or pseudoexfoliation syndrome were reported in at least three independent datasets. Therefore, a meta-analysis was not conducted for these metabolites due to insufficient data.

## Discussion

We conducted this meta-analysis to address the need for a better understanding of metabolomic alterations in glaucoma. We identified several pathways and metabolites in aqueous humour and plasma with a significant difference between patients with POAG and healthy controls. These results point towards underlying metabolic alterations in POAG which can help to identify early diagnostic biomarkers and novel therapies for POAG. The only previous meta-analysis of metabolomics in glaucoma was able to assess six AH metabolites and five plasma metabolites.^17^ With additional metabolomics studies conducted since 2021, we were able to significantly expand on the scope of our meta-analysis and assess a total of 19 metabolites in AH and eight metabolites in plasma, each reported in at least three independent datasets. Our results confirm previous findings of differences in AH lysine and alanine in POAG and identify glutamine, histidine, carnitine and creatinine as novel AH metabolites favouring POAG. We also confirm the previous finding of increased methionine in POAG plasma.^17^

A key finding is the enrichment of the arginine and proline metabolism pathway in both plasma and AH of patients with POAG. This finding aligns with previous studies that have shown significant enrichment of this pathway in these patients, indicating a potential role in POAG pathology.^17^ Arginine and proline metabolism is involved in nitric oxide production, which regulates vascular function and IOP, as well as collagen synthesis, which is essential for maintaining structural integrity.

Phenylalanine metabolism was also identified as an enriched metabolic pathway in the plasma of patients with POAG. Phenylalanine is metabolised into tyrosine, which in turn is converted into neurotransmitters such as dopamine, norepinephrine and epinephrine. ROS are generated during these metabolic steps,^20^ and an overactive phenylalanine pathway may contribute to oxidative stress. Enrichment of phenylalanine metabolism has been observed in conditions associated with systemic inflammation and oxidative stress, including type 2 diabetes^21^ and sepsis.^22^ Enrichment of this pathway could potentially serve as a marker for these underlying processes. However, in our study, neither phenylalanine nor tyrosine levels, which are part of the pathway, were significantly increased in plasma or AH. Further examination of AH metabolites within phenylalanine metabolism may provide additional insights into the pathophysiology of POAG.

Understanding both enrichment and impact is essential for interpreting the clinical relevance of any metabolic change. The glycine, serine and threonine pathway had significant enrichment in plasma, but also had an impact value of zero. This suggests that despite differential expression of several metabolites in this pathway, its overall functional significance may be limited. With only six AH metabolites and one plasma metabolite found to be significantly altered (from 19 metabolites in AH and eight metabolites in plasma), there are no pathways predicted to be significantly altered, although this is likely due to a small data set. This highlights the need for continued metabolomics studies and subsequent meta-analyses to predict pathway changes with confidence.

We also observed increased levels of metabolites such as lysine, alanine, glutamine, histidine, carnitine and creatinine in the AH of patients with POAG. Lysine, an essential amino acid important for protein synthesis and immune function, is crucial for collagen cross-linking. Deficiency in lysine can compromise collagen’s structural integrity. Given that POAG is associated with structural changes in the ONH^23^ and trabecular meshwork,^24^ where collagen fibres provide structural support,^25^ higher lysine levels may represent a compensatory mechanism for tissue repair. Similarly, increased levels of carnitine in AH may reflect a response to mitochondrial dysfunction and oxidative stress associated with POAG,^26^ as carnitine is essential for transporting long-chain fatty acids into mitochondria for β-oxidation and ATP production.^27^

Glutamine levels were significantly elevated in the AH of patients with POAG, without a corresponding increase in glutamate levels or significant changes in plasma glutamine. Glutamine is a precursor to glutamate, the primary excitatory neurotransmitter in the central nervous system.^28^ While glutamate excitotoxicity has been proposed as a mechanism of RGC death in glaucoma, as excessive glutamate can overactivate N-methyl-D-aspartate (NMDA)-receptors,^29^ leading to calcium overload and neuronal death,^30^ this hypothesis has been challenged. Studies have failed to detect elevated vitreous glutamate concentrations in glaucoma patients^31^ or animal models.^32^ In addition, two phase 3 studies on memantine, an NMDA-receptor antagonist which prevents excessive calcium influx, did not prevent glaucomatous progression in patients with open-angle glaucoma.^33^ Our findings suggest that localised alterations in AH glutamine metabolism may not translate into increased glutamate levels, or that compensatory mechanisms prevent glutamate accumulation.

Methionine was the only metabolite significantly increased in the plasma of patients with POAG. It is converted into S-adenosylmethionine, a methyl donor essential for DNA methylation and repair.^34^ Lower systemic levels of glutathione (GSH) have been reported in patients with POAG,^35^ potentially due to increased oxidative stress and deregulation of metabolic pathways responsible for GSH synthesis. The elevated methionine levels may reflect metabolic adaptations related to oxidative stress management.

In summary, our findings highlight specific metabolic pathways and metabolites altered in POAG. These metabolic changes may contribute to the disease’s pathophysiology and could serve as potential biomarkers or therapeutic targets. More research is needed to better understand how these metabolic changes contribute to the progression of POAG and to investigate how they might be used in clinical settings for diagnosis or treatment.

### Limitations

Substantial heterogeneity among studies complicated the interpretation of pooled results, likely due to differences in analytical methods, study designs and population factors. Variations in techniques like LC-MS, GC-MS and NMR further impacted metabolite detectability and quantification. Most studies did not adjust for disease stages, comorbidities or medications, all of which could influence metabolite profiles. The included studies were largely cross-sectional, providing only a single-timepoint metabolic snapshot. Longitudinal studies are needed to track changes, establish causal relationships and assess diagnostic biomarkers. Additionally, many studies used semi-targeted methods with predefined metabolite panels, limiting insights into systemic changes in POAG. This highlights the need for longitudinal studies, comprehensive phenotyping, and untargeted profiling to better understand metabolic alterations in POAG.

## Conclusions

This meta-analysis identified significant metabolic alterations associated with POAG, notably the enrichment of key metabolic pathways and the elevation of specific metabolites in aqueous humour and plasma. These findings underscore metabolic dysfunction as a central factor in POAG pathology, highlighting its potential for the development of biomarkers for early diagnosis and the creation of targeted therapies for glaucoma. Future research should focus on longitudinal studies and untargeted metabolomic profiling to deepen our understanding of underlying metabolic changes in POAG.

## supplementary material

10.1136/bmjophth-2024-002103 online supplemental file 1

10.1136/bmjophth-2024-002103 online supplemental file 2

10.1136/bmjophth-2024-002103 online supplemental file 3

10.1136/bmjophth-2024-002103 online supplemental file 4

## Data Availability

All data relevant to the study are included in the article or uploaded as supplementary information.
