# Supplementary material for: Identifying potential key metabolic pathways and biomarkers in glaucoma: a systematic review and meta-analysis
Source: BMJ Open Ophthalmol. 2025 Mar 13;10(1):e002103. doi: 10.1136/bmjophth-2024-002103 (PMC11907043; doi:10.1136/bmjophth-2024-002103)
Supplement: online supplemental file 1 [file bmjophth-10-1-s001.docx]

**Appendix A.** Table 1 with references.

| References | **First Author (Year)** | **Country** | **Glaucoma Type (n)** | **Control**  **Type (n)** | **Sample type** | **Technique** |
| --- | --- | --- | --- | --- | --- | --- |
| (1) | Barbosa Breda (2020) | Belgium | POAG (27), NTG (27) | Cataract (29) | AH | 1H NMR |
| (2) | Botello-Marabotto (2024) | Spain | POAG (11) | Controls (19) | Tears | 1H-NMR |
| (3) | Buisset (2019) | France | POAG (26) | Cataract (26) | AH | LC-MS |
| (4) | Kang (2022) | USA | XFG (205) | Controls (205) | Plasma | LC-MS |
| (5) | Nzoughet (2020) | France | POAG (34) | Cataract (30) | Plasma | LC-HRMS |
| (6) | Leruez (2018) | France | POAG (36) | Cataract (27) | Plasma | LC-MS/MS |
| (7) | Li (2024) | China | PACG (348) | Controls (268) | Serum | UPLC |
| (8) | Lillo (2022) | Spain | OAG (8) | Refractive error (16) | AH | LC-MS/MS |
| (9) | Myer (2020) | USA | POAG (16), PEX (31) | Controls (25) | AH | For POAG: 1H-NMR, For PEX: IROA  LC-MS. |
| (10) | Pan (2020) | China | POAG (16) | Cataract (24) | AH | GC-TOF-MS |
| (11) | Pulukool (2021) | India | POAG (20) | Cataract (20) | AH | GC-TOF-MS |
| (12) | Rossi (2019) | Italy | POAG (16) | Controls (17) | Tears | LC-MS/MS |
| (13) | Tang (2021) | China | POAG (25) | Cataract (25) | AH + Plasma | LC-MS/MS |
| (14) | Zeleznik (2023) | USA | POAG (599) | Controls (599) | Plasma | LC-MS/MS + NMR |
| (15) | Gowtham (2023) | India | POAG (14) + PACG (14) | Cataract (14) | AH + Plasma | LC-HRMS |
| (16) | Gong (2020) | China | POAG (30) | Controls (30) | Serum | GC-MS |
| (17) | Burgess (2015) | USA | POAG (72) | Controls (72) | Plasma | LC-MS |

1. Barbosa Breda J, Croitor Sava A, Himmelreich U, Somers A, Matthys C, Rocha Sousa A, m.fl. Metabolomic profiling of aqueous humor from glaucoma patients - The metabolomics in surgical ophthalmological patients (MISO) study. Experimental Eye Research. 12e.Kr.;201:108268.

2. Botello-Marabotto M, Martinez-Bisbal MC, Pinazo-Duran MD, Martinez-Manez R. Tear metabolomics for the diagnosis of primary open-angle glaucoma. Talanta. 25 februari ;273:125826.

3. Buisset A, Gohier P, Leruez S, Muller J, Amati-Bonneau P, Lenaers G, m.fl. Metabolomic Profiling of Aqueous Humor in Glaucoma Points to Taurine and Spermine Deficiency: Findings from the Eye-D Study. Journal of Proteome Research. 03 01;18(3):1307–15.

4. Kang JH, Zeleznik O, Frueh L, Lasky-Su J, Eliassen AH, Clish C, m.fl. Prediagnostic Plasma Metabolomics and the Risk of Exfoliation Glaucoma. Investigative Ophthalmology & Visual Science. 08 02;63(9):15.

5. Kouassi Nzoughet J, Guehlouz K, Leruez S, Gohier P, Bocca C, Muller J, m.fl. A Data Mining Metabolomics Exploration of Glaucoma. Metabolites. 28 januari 2020;10(2):49.

6. Leruez S, Marill A, Bresson T, e Saint Martin G, Buisset A, Muller J, m.fl. A Metabolomics Profiling of Glaucoma Points to Mitochondrial Dysfunction, Senescence, and Polyamines Deficiency. Investigative Ophthalmology & Visual Science. 09 04;59(11):4355–61.

7. Li S, Ren J, Jiang Z, Qiu Y, Shao M, Li Y, m.fl. Metabolomics identifies and validates serum androstenedione as novel biomarker for diagnosing primary angle closure glaucoma and predicting the visual field progression. eLife. 15 februari ;12:15.

8. Lillo A, Marin S, Serrano-Marin J, Binetti N, Navarro G, Cascante M, m.fl. Targeted Metabolomics Shows That the Level of Glutamine, Kynurenine, Acyl-Carnitines and Lysophosphatidylcholines Is Significantly Increased in the Aqueous Humor of Glaucoma Patients. Frontiers in Medicine. 2022;9:935084.

9. Myer C, Abdelrahman L, Banerjee S, Khattri RB, Merritt ME, Junk AK, m.fl. Aqueous humor metabolite profile of pseudoexfoliation glaucoma is distinctive. Molecular Omics. 10 12;16(5):425–35.

10. Pan CW, Ke C, Chen Q, Tao YJ, Zha X, Zhang YP, m.fl. Differential metabolic markers associated with primary open-angle glaucoma and cataract in human aqueous humor. BMC Ophthalmology. maj ;20(1):183.

11. Pulukool SK, Bhagavatham SKS, Kannan V, Sukumar P, Dandamudi RB, Ghaisas S, m.fl. Elevated dimethylarginine, ATP, cytokines, metabolic remodeling involving tryptophan metabolism and potential microglial inflammation characterize primary open angle glaucoma. Scientific Reports. 05 07;11(1):9766.

12. Rossi C, Cicalini I, Cufaro MC, Agnifili L, Mastropasqua L, Lanuti P, m.fl. Multi-Omics Approach for Studying Tears in Treatment-Naive Glaucoma Patients. International Journal of Molecular Sciences. 18 augusti ;20(16):18.

13. Tang Y, Pan Y, Chen Y, Kong X, Chen J, Zhang H, m.fl. Metabolomic Profiling of Aqueous Humor and Plasma in Primary Open Angle Glaucoma Patients Points Towards Novel Diagnostic and Therapeutic Strategy. Frontiers in Pharmacology. 2021;12:621146.

14. Zeleznik OA, Kang JH, Lasky-Su J, Eliassen AH, Frueh L, Clish CB, m.fl. Plasma metabolite profile for primary open-angle glaucoma in three US cohorts and the UK Biobank. Nature communications. 05 19;14(1):2860.

15. Gowtham L, Halder N, Angmo D, Singh SB, Jayasundar R, Dada T, m.fl. Untargeted metabolomics in the aqueous humor reveals the involvement of TAAR pathway in glaucoma. Experimental Eye Research [Internet]. 2023;234. Tillgänglig vid: https://www.embase.com/search/results?subaction=viewrecord&id=L2026367254&from=export http://dx.doi.org/10.1016/j.exer.2023.109592

16. Gong H, Zhang S, Li Q, Zuo C, Gao X, Zheng B, m.fl. Gut microbiota compositional profile and serum metabolic phenotype in patients with primary open-angle glaucoma. Experimental Eye Research. 2e.Kr.;191:107921.

17. Burgess LG, Uppal K, Walker DI, Roberson RM, Tran V, Parks MB, m.fl. Metabolome-Wide Association Study of Primary Open Angle Glaucoma. Investigative Ophthalmology & Visual Science. juli ;56(8):5020–8.
