# Supplementary material for: Identifying potential key metabolic pathways and biomarkers in glaucoma: a systematic review and meta-analysis
Source: BMJ Open Ophthalmol. 2025 Mar 13;10(1):e002103. doi: 10.1136/bmjophth-2024-002103 (PMC11907043; doi:10.1136/bmjophth-2024-002103)
Supplement: online supplemental file 2 [file bmjophth-10-1-s002.pdf]

1. Medline

Interface: Ovid MEDLINE(R) ALL<br><br>Date of Search: 15 March 2024<br><br>Number of hits: 1,039<br><br>Comment: In Ovid, two or more words are automatically searched as phrases; i.e. no quotation marks are needed	Field labels<br><br>• exp/ = exploded MeSH term<br>• / = non exploded MeSH term<br>• .ti,ab,kf. = title, abstract and author keywords<br>• adjx = within x words, regardless of order<br>• * = truncation of word for alternate endings

Database(s): **Ovid MEDLINE(R) ALL** 1946 to March 14, 2024
Search Strategy:

#	Searches	Results
1	exp Glaucoma/	60791
2	glaucoma*.ti,ab,kf.	73844
3	1 or 2	84454
4	exp Metabolomics/	28546
5	exp Metabolome/	16468
6	exp Glaucoma/bl	473
7	(metabol* or lipidom* or metabonomic*).ti,ab,kf.	1607230
8	or/4-7	1609871
9	3 and 8	1902
10	(animals not humans).sh.	5169143
11	9 not 10	1684
12	limit 11 to english language	1447
13	limit 12 to "review articles"	377
14	review.ti.	743520
15	(review or systematic review or editorial or letter or study guide).pt.	5324096
16	13 or 14 or 15	5514509
17	12 not 16	1039

Karolinska Institutet

2. Embase

Interface: embase.com<br><br>Date of Search: 15 March 2024<br><br>Number of hits: 974<br><br>Comment: Emtree is the controlled vocabulary in Embase	Field labels<br><br>• /exp = exploded Emtree term<br>• /de = non exploded Emtree term<br>• ti,ab,kw = title, abstract and author keywords<br>• NEAR/x = within x words, regardless of order<br>• * = truncation of word for alternate endings

No.	Query	Results
#12	#8 NOT #9 NOT ([animals]/lim NOT [humans]/lim) AND [english]/lim	974
#11	#8 NOT #9 NOT ([animals]/lim NOT [humans]/lim)	1149
#10	#8 NOT #9	1418
#9	#3 AND #7 AND ([conference abstract]/lim OR [conference paper]/lim OR [conference review]/lim OR [editorial]/lim OR [letter]/lim OR [review]/lim)	1173
#8	#3 AND #7	2591
#7	#4 OR #5 OR #6	1978709
#6	metabol*:ti,ab,kw OR lipidom*:ti,ab,kw OR metabonomic*:ti,ab,kw	1971379
#5	'metabolome'/exp	17646
#4	'metabolomics'/exp	64672
#3	#1 OR #2	127076
#2	glaucoma*:ti,ab,kw	94901
#1	'glaucoma'/exp	113485

Karolinska Institutet

4. Web of Science Core Collection

Interface: Clarivate Analytics<br><br>Editions = A&HCI , ESCI , SCI-EXPANDED , SSCI<br><br>Date of Search: 15 March 2024<br><br>Number of hits: 929	Field labels<br><br>• TS/Topic = title, abstract, author keywords and Keywords Plus<br>• NEAR/x = within x words, regardless of order<br>• * = truncation of word for alternate endings<br><br>Note: the *Exact search*-function was used for all the searches

#	Search Query	Results
1	TS=glaucoma*	78109
2	TS=(metabol* or lipidom* or metabonomic*)	1951239
3	#2 AND #1	1674
4	#2 AND #1 and Article or Early Access (Document Types)	1220
5	#2 AND #1 and Article or Early Access (Document Types) and Article or Early Access (Document Types)	1220
6	#2 AND #1 and Article or Early Access (Document Types) and Article or Early Access (Document Types) and English (Languages)	1177
7	TS=((animal* OR rat OR rats OR mouse OR mice OR murine OR dog OR dogs OR canine OR cat OR cats OR feline OR rabbit OR cow OR cows OR bovine OR rodent* OR sheep OR ovine OR pig OR swine OR porcine OR veterinar* OR chick* OR zebrafish* OR baboon* OR nonhuman* OR primate* OR cattle* OR goose OR geese OR duck OR macaque* OR avian* OR bird* OR fish*) NOT (human* OR patient* OR women OR woman OR men OR man))	5343371
8	#6 NOT #7	929
