# Supplementary material for: Identifying potential key metabolic pathways and biomarkers in glaucoma: a systematic review and meta-analysis
Source: BMJ Open Ophthalmol. 2025 Mar 13;10(1):e002103. doi: 10.1136/bmjophth-2024-002103 (PMC11907043; doi:10.1136/bmjophth-2024-002103)

**Supplementary Figure 1-2**

**Supplementary Figure 1**. Forest plot of the non-significant metabolites in plasma of POAG patients compared to control participants. Individual and pooled analysis presented as effect size (log[OR]) and CI 95% of nicotinamide, arginine, glutamine, phenylalanine, proline, tyrosine, hydroxyproline, xanthine and leucine.


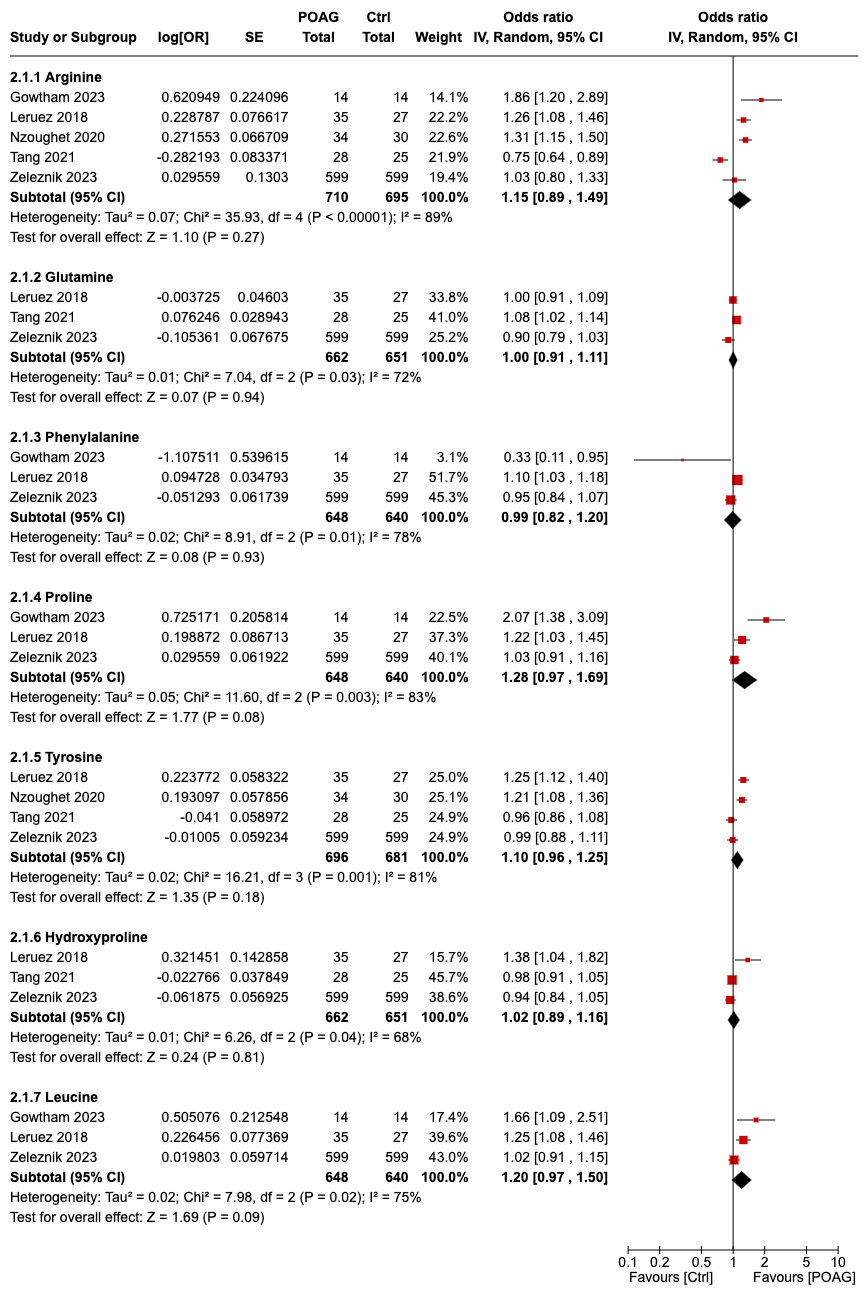


**Supplementary Figure 2.** Forest plot of the non-significant metabolites in aqueous humor of POAG patients compared to control participants. Individual and pooled analysis presented as effect size (log[OR]) and CI 95% of lactic acid, propionylcarnitine, creatine, phenylalanine, valine, glutamic acid, glycine, leucine, isoleucine, tyrosine, tryptophan, arginine.


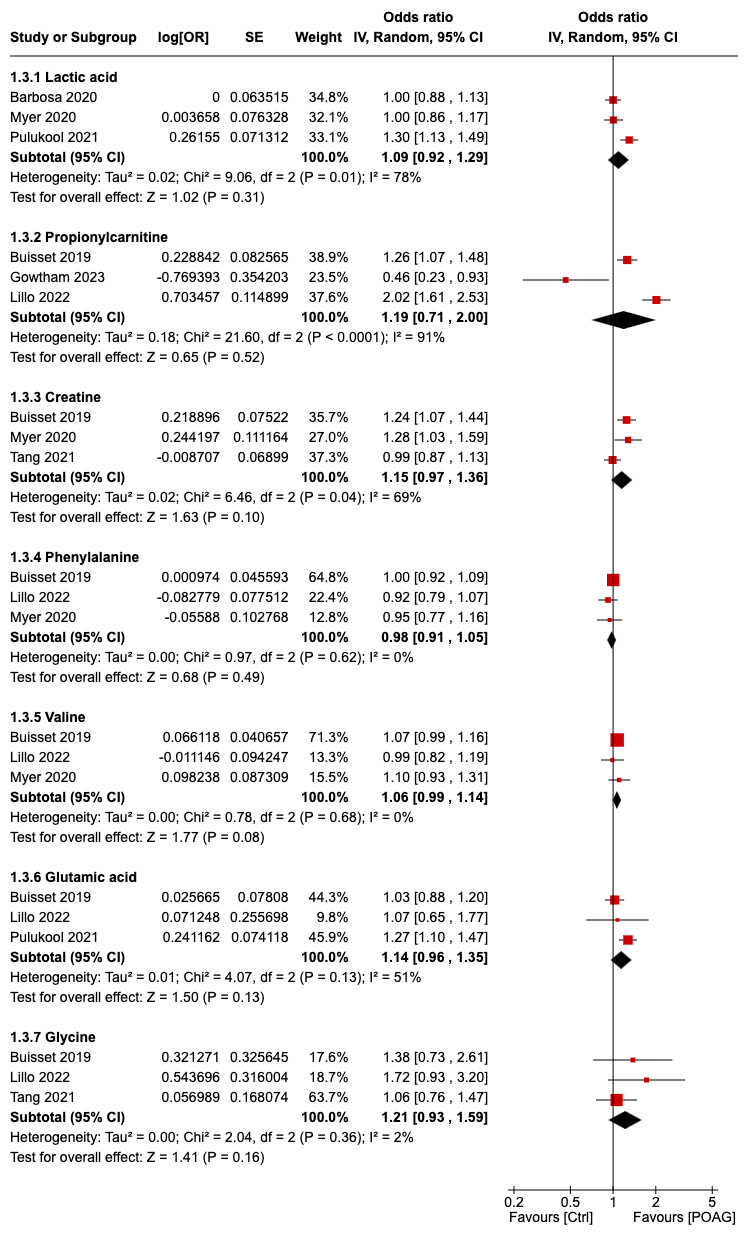


**Supplementary Figure 2 (continued).**


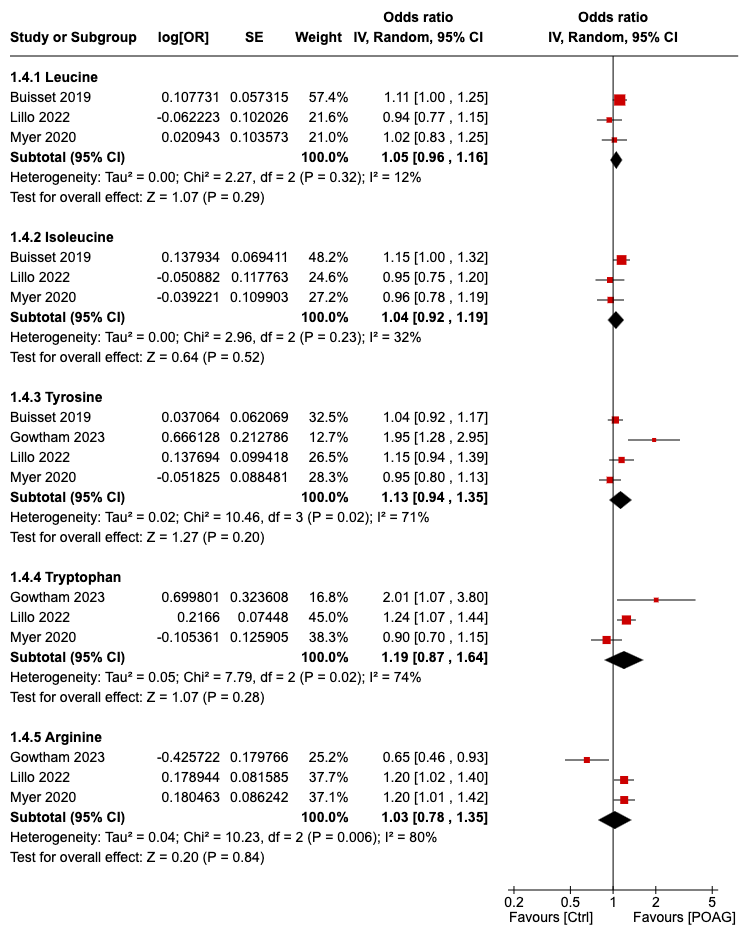

Supplement: online supplemental file 3 [file bmjophth-10-1-s003.docx]
