# Supplementary material for: Identifying potential key metabolic pathways and biomarkers in glaucoma: a systematic review and meta-analysis
Source: BMJ Open Ophthalmol. 2025 Mar 13;10(1):e002103. doi: 10.1136/bmjophth-2024-002103 (PMC11907043; doi:10.1136/bmjophth-2024-002103)
Supplement: online supplemental file 4 [file bmjophth-10-1-s004.docx]

**Supplementary Table 1-4**

**Supplementary Table 1.** Heterogeneity classification according to Cochrane Collaboration.

| Grading | I^2^ |
| --- | --- |
| Low heterogeneity | 0-40% |
| Moderate heterogeneity | 30-60% |
| Substantial heterogeneity | 50-90% |
| Considerable heterogeneity | 75-100% |

**Supplementary Table 2.** Methodological quality assessment using the QUADOMICS tool.

| First Author (Year) | **1** | **2** | **3** | **4** | **5** | **6** | **7** | **8** | **9** | **10** | **11** | **12** | **13** | **14** | **15** | **16** | **Score** |
| --- | --- | --- | --- | --- | --- | --- | --- | --- | --- | --- | --- | --- | --- | --- | --- | --- | --- |
| Barbosa Breda (2020) | Y | Y | Y | Y | Y | Y | Y | Y | Y | Y | Y | Y | Y | Y | Y | Y | 16/16 |
| Botello-Marabotto (2024) | Y | Y | Y | Y | Y | Y | Y | Y | Y | Y | Y | Y | Y | Y | Y | Y | 16/16 |
| Buisset (2019) | Y | Y | Y | Y | Y | Y | Y | Y | Y | Y | Y | N/A | N/A | Y | Y | Y | 14/14 |
| Kang (2022) | Y | N | Y | Y | Y | N/A | Y | Y | Y | Y | Y | Y | Y | Y | N | Y | 14/15 |
| Nzoughet (2020) | Y | Y | Y | Y | Y | N/A | Y | Y | Y | Y | Y | N/A | N/A | Y | N/A | Y | 12/12 |
| Leruez (2018) | Y | Y | Y | Y | Y | N/A | Y | Y | Y | Y | Y | N/A | N/A | Y | N/A | Y | 12/12 |
| Li  (2024) | Y | Y | Y | Y | Y | Y | Y | Y | Y | Y | Y | Y | Y | Y | Y | Y | 16/16 |
| Lillo  (2022) | Y | Y | Y | Y | Y | N/A | Y | Y | Y | Y | Y | N/A | N/A | Y | N/A | Y | 12/12 |
| Myer (2020) | Y | Y | Y | Y | Y | Y | Y | Y | Y | Y | Y | Y | Y | Y | N | Y | 15/16 |
| Pan  (2020) | Y | Y | Y | Y | Y | Y | Y | Y | Y | Y | Y | N/A | N/A | Y | N | Y | 13/14 |
| Pulukool (2021) | Y | Y | Y | Y | Y | Y | Y | Y | Y | Y | Y | Y | Y | Y | N | Y | 15/16 |
| Rossi (2019) | Y | Y | Y | Y | Y | Y | Y | Y | Y | Y | Y | Y | Y | Y | N | Y | 15/16 |
| Tang (2021) | Y | Y | Y | Y | Y | Y | Y | Y | Y | Y | Y | Y | Y | Y | N | Y | 15/16 |
| Zeleznik (2023) | Y | Y | Y | Y | Y | Y | Y | Y | Y | Y | Y | Y | Y | Y | Y | Y | 16/16 |
| Gowtham (2023) | Y | Y | Y | Y | Y | Y | Y | Y | Y | Y | Y | Y | Y | Y | N/A | Y | 15/15 |
| Gong (2020) | Y | Y | Y | Y | Y | Y | Y | Y | Y | Y | Y | Y | Y | Y | Y | Y | 16/16 |
| Burgess (2015) | Y | Y | Y | Y | Y | Y | Y | Y | Y | Y | Y | Y | Y | Y | N | Y | 15/16 |

Footnote: The table is based on the following criteria, graded as Y = criteria achieved, N = criteria not achieved, N/A = not applicable.

1. Were selection criteria clearly described?

2. Was the spectrum of patients representative of patients who will receive the test in practice?

3. Was the type of sample fully described?

4. Were the procedures and timing of biological sample collection with respect to clinical factors described with enough detail?

4.1. Clinical and physiological factors

4.2. Diagnostic and treatment procedures.

5. Were handling and pre-analytical procedures reported in sufficient detail and similar for the whole sample? and, if differences in procedures were reported, was their effect on the results assessed?

6. Is the time period between the reference standard and the index test short enough to reasonably guarantee that the target condition did not change between the two tests?

7. Is the reference standard likely to correctly classify the target condition?

8. Did the whole sample or a random selection of the sample receive verification using a reference standard of diagnosis?

9. Did patients receive the same reference standard regardless of the result of the index test?

10. Was the execution of the index test described in sufficient detail to permit replication of the test?

11. Was the execution of the reference standard described in sufficient detail to permit its replication?

12. Were the index test results interpreted without knowledge of the results of the reference standard?

13. Were the reference standard results interpreted without knowledge of the results of the index test?

14. Were the same clinical data available when test results were interpreted as would be available when the test is used in practice?

15. Were uninterpretable/intermediate test results reported?

16. Is it likely that the presence of overfitting was avoided?

**Supplementary Table 3.** Repeated significant metabolic biomarkers associated with POAG.

| Metabolite | **HMDB ID** | **Frequency** | **Difference** | **Sample** | **Subject** |
| --- | --- | --- | --- | --- | --- |
| Hypoxanthine | HMDB0000157 | 5 | Up | AH | Pulukool (2021) |
|  |  |  |  | Serum | Gong (2020) |
|  |  |  |  | Plasma | Tang (2021) |
|  |  |  | Down | AH | Tang (2021) |
|  |  |  |  | Plasma | Nzoughet (2020) |
| Arginine | HMDB0000517 | 5 | Up | AH | Lillo (2022) |
|  |  |  |  | Plasma | Gowtham (2023),  Nzoughet (2020) |
|  |  |  | Down | AH | Gowtham (2023) |
| Alanine | HMDB0000517 | 4 | Up | AH | Barbosa Breda (2020),  Lillo (2022),  Pulukool (2021) |
|  |  |  | Down |  | Buisset (2019) |
| Propionyl-carnitine | HMDB0000824 | 4 | Up | AH | Lillo (2022) |
|  |  |  |  | Plasma | Leruez (2018) |
|  |  |  | Down | AH | Buisset (2019),  Gowtham (2023) |
| Methionine | HMDB0000214 | 4 | Up | AH | Lillo (2022) |
|  |  |  |  | Plasma | Nzoughet (2020),  Gowtham (2023),  Lereuz (2018) |
| Lysine | HMDB0000696 | 3 | Up | AH | Lillo (2022),  Pulukool (2021) |
|  |  |  |  | Serum | Gong (2020) |

**Supplementary Table 3.** Repeated significant metabolic biomarkers associated with POAG (continued).

| Metabolite | **HMDB ID** | **Frequency** | **Difference** | **Sample** | **Subject** |
| --- | --- | --- | --- | --- | --- |
| Glutamine | HMDB0000182 | 3 | Up | AH | Pulukool (2021) |
|  |  |  | Down |  | Buisset (2019),  Lillo (2022) |
| Nicotinamide | HMDB0001406 | 3 | Down | Plasma | Nzoughet (2020) |
|  |  |  |  | AH | Pulukool (2021) |
| Tyrosine | HMDB0000158 | 3 | Up | Plasma | Leruez (2018),  Nzoughet (2020) |
|  |  |  | Down | AH | Gowtham (2023) |
| Spermidine | HMDB0000725 | 3 | Up | AH | Lillo (2022) |
|  |  |  | Down | Plasma | Lereuz (2018) |
|  |  |  |  | AH | Pan (2020) |

Abbreviations: AH, Aqueous humor.

**Supplementary Table 4.** Metabolic pathway analysis in MetaboAnalyst 5.0 was conducted using all upregulated metabolites in both the aqueous humor (n = 33) and plasma (n = 54) of POAG patients compared to controls.

| Sample type | Pathway | Enrichment analysis | | | Topology analysis |
| --- | --- | --- | --- | --- | --- |
|  |  | **Match Status** | **P-value** | **FDR** | **Impact** |
| Aqueous humor | Arginine and proline metabolism | 4/36 | 2.14E-4 | 0.0171 | 0.37558 |
| Plasma | Arginine and proline metabolism | 6/36 | 2.65E-4 | 0.0212 | 0.18721 |
| Plasma | Phenylalanine metabolism | 3/8 | 8.97E-4 | 0.0359 | 0.59524 |
| Plasma | Glycine, serine and threonine metabolism | 5/33 | 1.43E-3 | 0.0385 | 0.00000 |
